# First-line pembrolizumab + chemotherapy in Japanese patients with advanced/metastatic esophageal cancer from KEYNOTE-590

**DOI:** 10.1007/s10388-022-00920-x

**Published:** 2022-06-07

**Authors:** Takashi Kojima, Hiroki Hara, Akihito Tsuji, Hisateru Yasui, Kei Muro, Taroh Satoh, Takashi Ogata, Ryu Ishihara, Masahiro Goto, Hideo Baba, Tomohiro Nishina, Shirong Han, Tomoko Sakata, Naoyoshi Yatsuzuka, Toshihiko Doi, Ken Kato

**Affiliations:** 1 Department of Gastroenterology and Gastrointestinal Oncology, National Cancer Center Hospital East, Chiba, Japan; 2 Department of Gastroenterology, Saitama Cancer Center, Saitama, Japan; 3 Department of Medical Oncology, Kagawa University Hospital, Kagawa, Japan; 4 Department of Medical Oncology, Kobe City Medical Center General Hospital, Kobe, Japan; 5 Department of Clinical Oncology, Aichi Cancer Center Hospital, Aichi, Japan; 6 Department of Frontier Science for Cancer and Chemotherapy, Osaka University Hospital, Osaka, Japan; 7 Department of Gastrointestinal Surgery, Kanagawa Cancer Center, Yokohama, Japan; 8 Department of Gastrointestinal Oncology, Osaka International Cancer Institute, Osaka, Japan; 9 Cancer Chemotherapy Center, Osaka Medical and Pharmaceutical University Hospital, Osaka, Japan; 10 Department of Gastroenterological Surgery, Kumamoto University Hospital, Kumamoto, Japan; 11 Department of Gastrointestinal Medical Oncology, National Hospital Organization Shikoku Cancer Center, Ehime, Japan; 12 Department of Medical Oncology, MSD K.K, Tokyo, Japan; 13 Department of Gastrointestinal Oncology, National Cancer Center Hospital East, Chiba, Japan; 14 Department of Gastrointestinal Medical Oncology, National Cancer Center Hospital, Tokyo, Japan

**Keywords:** Esophageal squamous cell carcinoma, Immune checkpoint inhibitors, Immunotherapy, Pembrolizumab, Programmed death ligand 1

## Abstract

**Background:**

The phase 3 KEYNOTE-590 (NCT03189719) study showed first-line pembrolizumab plus chemotherapy significantly prolonged overall survival and progression-free survival versus placebo plus chemotherapy in patients with advanced unresectable or metastatic adenocarcinoma or squamous cell carcinoma of the esophagus or advanced/metastatic Siewert type I adenocarcinoma of the esophagogastric junction. We describe a subgroup analysis of Japanese patients from KEYNOTE-590.

**Methods:**

Eligible patients were randomly assigned 1:1 to pembrolizumab 200 mg intravenously every 3 weeks or placebo plus chemotherapy (cisplatin 80 mg/m^2^ and 5-fluorouracil 800 mg/m^2^/day). Efficacy was evaluated in all Japanese patients and those with esophageal squamous cell carcinoma and programmed death ligand 1 combined positive score ≥ 10. Dual primary endpoints were overall survival and progression-free survival per RECIST v1.1 by investigator. Secondary endpoints included objective response rate per RECIST v1.1 by investigator and safety and tolerability.

**Results:**

At data cutoff (July 2, 2020), 141 Japanese patients were randomly assigned (pembrolizumab plus chemotherapy, 74; placebo plus chemotherapy, 67). In all Japanese patients, median overall survival was 17.6 months with pembrolizumab plus chemotherapy versus 11.7 months with chemotherapy (hazard ratio, 0.71; 95% confidence interval, 0.47–1.09), median progression-free survival was 6.3 versus 6.0 months (hazard ratio, 0.58; 95% confidence interval, 0.40–0.84), and objective response rate was 56.8% versus 38.8%. Grade 3–5 treatment-related adverse events were 74.3% and 61.2%.

**Conclusion:**

First-line pembrolizumab plus chemotherapy demonstrated improvement in overall survival and progression-free survival compared with placebo plus chemotherapy in Japanese patients with advanced/metastatic esophageal cancer; safety was comparable between treatment groups.

**Clinical trial registry:**

ClinicalTrials.gov, NCT03189719.

**Supplementary Information:**

The online version contains supplementary material available at 10.1007/s10388-022-00920-x.

## Introduction

In 2018, 20,000 cases and 12,000 deaths from esophageal cancer (EC) were reported in Japan [1]. The two primary subgroups of EC are esophageal squamous cell carcinoma (ESCC) and adenocarcinoma, and they vary in etiology and geographic distribution. ESCC is the predominant type of EC in East Asia, including Japan, where the ratio of ESCC to adenocarcinoma is 26:1 [2].

Practice guidelines in Japan recommend cisplatin plus 5-fluoruracil (5-FU) as first-line therapy for unresectable advanced or recurrent EC [3]. The median duration of survival for Japanese patients with advanced EC receiving chemotherapy is < 8.1 months, highlighting the unmet need for these patients [4]. Pembrolizumab is approved in Japan for the treatment of patients with radically unresectable advanced or recurrent ESCC of the esophagus whose tumors express PD-L1 (combined positive score [CPS] ≥ 10) with disease progression after ≥ 1 line of chemotherapy based on data from the phase 3 KEYNOTE-181 study [5–7].

KEYNOTE-590 [7] was a randomized double-blind study of first-line pembrolizumab plus chemotherapy (pembrolizumab–chemotherapy) versus placebo plus chemotherapy (placebo–chemotherapy) in patients with advanced EC [8, 9]; 749 patients were enrolled regardless of PD-L1 status [9]. Pembrolizumab–chemotherapy was superior to placebo–chemotherapy for overall survival (OS) in the total population (hazard ratio [HR], 0.73; *P* < 0.0001) and in patients with ESCC (HR, 0.72; *P* = 0.0006), PD-L1 CPS ≥ 10 (HR, 0.62; *P* < 0.0001), and ESCC PD-L1 CPS ≥ 10 (HR, 0.57; *P* < 0.0001) [9]. Pembrolizumab–chemotherapy was superior to placebo–﻿chemotherapy for progression-free survival (PFS) (*P* < 0.0001) in the total population and in patients with ESCC and PD-L1 CPS ≥ 10. Objective response rate (ORR) was 45.0% in patients treated with pembrolizumab–chemotherapy compared with 29.3% in patients treated with placebo–chemotherapy (*P* < 0.0001) [9].

Data from KEYNOTE-590 in patients with untreated advanced esophageal and esophagogastric junction (EGJ) cancer demonstrated that first-line pembrolizumab–chemotherapy is a new standard of care. Herein, we present the results in the Japanese population of the KEYNOTE-590 study.

## Methods

### Study design, treatment, and participants

The study design of the randomized, double-blind, phase 3 KEYNOTE-590 trial has been published [7–9]. Briefly, eligible patients had treatment-naive, histologically or cytologically confirmed, locally advanced unresectable or metastatic esophageal adenocarcinoma, ESCC, or locally advanced or metastatic Siewert type I adenocarcinoma of the EGJ. Previous treatment with curative intent, including neoadjuvant or adjuvant treatment, was permissible if disease progression occurred > 6 months after cessation of treatment. Patients were randomly assigned 1:1 to receive intravenous (IV) pembrolizumab 200 mg or placebo (normal saline) every 3 weeks (Q3W) for up to 35 cycles (~ 2 years) plus chemotherapy (cisplatin 80 mg/m^2^ IV Q3W for ≤ 6 doses and 5-FU 800 mg/m^2^/day continuous IV infusion on days 1–5 Q3W per local standard) until disease progression, unacceptable toxicity, or withdrawal of consent. Randomization was stratified by geographic region (Asia vs. non-Asia), histology (adenocarcinoma vs. ESCC), and Eastern Cooperative Oncology Group (ECOG) performance status (0 vs. 1).

### Outcomes and assessments

Assessments of primary efficacy and safety outcomes have been described [8, 9]. In the current analysis, efficacy and safety endpoints were assessed in patients enrolled at Japanese sites. Dual primary endpoints were OS and PFS per RECIST v1.1 by investigator assessment. Secondary endpoints included ORR and duration of response (DOR) per RECIST v1.1 by investigator assessment, safety and tolerability, and health-related quality of life.

PD-L1 expression was assessed in archival or newly collected tumor samples using PD-L1 IHC 22C3 pharmDx (Agilent) and measured using CPS (defined as the number of PD-L1–staining cells [tumor cells, lymphocytes, macrophages] divided by the total number of viable tumor cells, multiplied by 100).

### Statistical analysis

In the Japanese population, efficacy was evaluated in the intention-to-treat population and in the ESCC, PD-L1 CPS ≥ 10, and ESCC PD-L1 CPS ≥ 10 subgroups as specified in the protocol. OS and PFS were estimated using the nonparametric Kaplan–Meier method, and treatment differences were assessed using a Cox proportional hazards model with Efron’s method of tie handling to estimate the magnitude of the treatment difference (HR). The Japanese subgroup analysis was not controlled for multiplicity, and no alpha was allocated to the comparisons. The estimated sample size of the Japanese population was calculated to guarantee that there is >80% probability of consistency between overall and Japanese populations on the primary endpoint of OS. Consistency was defined as the probability that the estimated HRs for the overall and Japanese populations are both < 1. The necessary sample size to achieve >80% probability of consistency in the subgroup of patients with ESCC PD-L1 CPS ≥ 10 was 55 and 45 for patients with ESCC. With 141 Japanese patients enrolled into the study, the probabilities increased to 91.6% for patients with ESCC PD-L1 CPS ≥ 10 and 93.6% for patients with ESCC.

Data cutoff for protocol-specified interim OS and final PFS analyses was July 2, 2020. This trial is registered with ClinicalTrials.gov (NCT03189719).

## Results

### Patients

Of 749 patients enrolled in KEYNOTE-590, 141 were in Japan (pembrolizumab–chemotherapy, 74; placebo–chemotherapy, 67) (Online Resource 1). Baseline characteristics were generally well balanced between treatment groups (Table 1). Most patients had metastatic disease at baseline in both the pembrolizumab–chemotherapy group (90.5%) and the placebo–chemotherapy group (88.1%). The most common locations for metastasis for Japanese patients in both the pembrolizumab–chemotherapy group and placebo–chemotherapy group were lymph node (74.3%; 71.6%), lung (28.4%; 23.9%), liver (24.3%; 20.9%), abdominal lymph node (20.3%; 19.4%), and bone (9.5%; 13.4%). More patients in the pembrolizumab–chemotherapy group than in the placebo–chemotherapy group had ECOG performance status 1 (35.1% vs. 20.9%) and PD-L1 CPS ≥ 10 (64.9% vs. 53.7%). Median time from randomization to date of death or data cutoff was 24.4 months (range, 17.6–33.4). At the time of data cutoff, most patients had discontinued study treatment (pembrolizumab–chemotherapy, 64 [86.5%]; placebo–chemotherapy, 65 [97.0%]) (Online Resource 1). Treatment was discontinued in 43/74 patients (58.1%) in the pembrolizumab–chemotherapy group and 53/67 patients (79.1%) in the placebo–chemotherapy treatment group because of progressive disease. Five patients (6.8%) treated with pembrolizumab–chemotherapy completed 35 treatment cycles (~ 2 years).

**Table 1:** Baseline patient demographics and disease characteristics of the Japanese population

Characteristic	Pembrolizumab + chemotherapy *n* = 74	Placebo + chemotherapy *n* = 67
Median age, years (range)	68 (32–81)	68 (46–79)
Male, *n* (%)	63 (85.1)	61 (91.0)
ECOG PS, *n* (%)		
0	48 (64.9)	53 (79.1)
1	26 (35.1)	14 (20.9)
Histology, *n* (%)		
ESCC	67 (90.5)	59 (88.1)
AC of esophagus or EGJ	7 (9.5)	8 (11.9)
PD-L1 CPS *n* (%)^a^		
≥ 10	48 (64.9)	36 (53.7)
< 10	21 (28.4)	30 (44.8)
Not evaluable/missing	5 (6.8)	1 (1.5)
Disease stage, *n* (%)		
Locally advanced	7 (9.5)	8 (11.9)
Metastatic	67 (90.5)	59 (88.1)
Previous therapy, *n* (%)		
No	36 (48.6)	38 (56.7)
Yes (recurrent after curative therapy)^a^	38 (51.4)	29 (43.3)
Previous radiation therapy, *n* (%)		
No	60 (81.1)	54 (80.6)
Yes	14 (18.9)	13 (19.4)

*AC* adenocarcinoma, *CPS* combined positive score, *ECOG PS* Eastern Cooperative Oncology Group performance status, *EGJ* esophagogastric junction, *ESCC* esophageal squamous cell carcinoma, *PD-L1* programmed death ligand 1

^a^Treatment with curative intent was permissible if disease progression occurred > 6 months after the cessation of treatment

Of patients who discontinued treatment, 44 of 64 patients (68.8%) in the pembrolizumab–chemotherapy group and 49 of 65 patients (75.4%) in the placebo–chemotherapy group received subsequent therapy (Online Resource 2). Paclitaxel was the most common in the pembrolizumab–chemotherapy group (31 of 64 patients; 48.4%) and the placebo–chemotherapy group (36 of 65 patients; 55.4%), and nivolumab was the most common subsequent immunotherapy received in 5 of 64 patients (7.8%) and 11 of 65 patients (16.9%), respectively.

### Overall survival

**Fig. 1:**
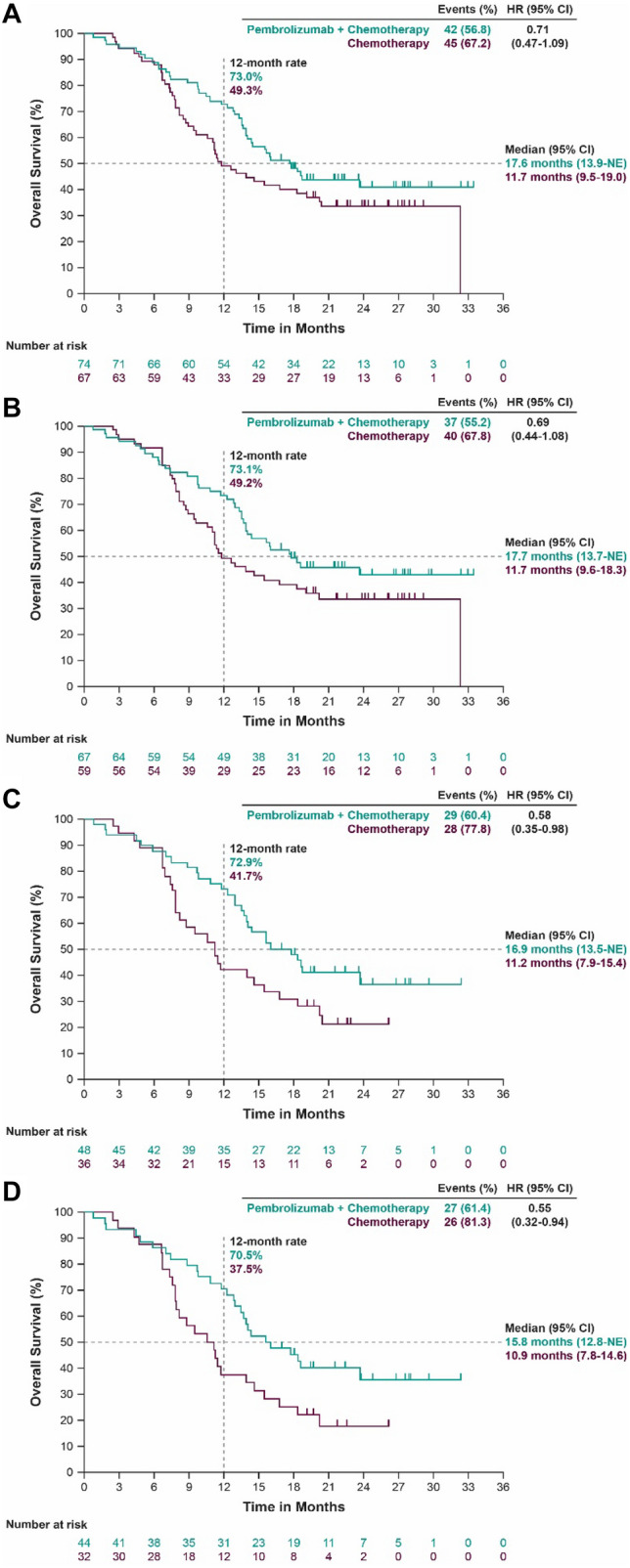
Kaplan–Meier estimates of overall survival in the Japanese population by treatment group. **a** All patients. **b** Patients with ESCC. **c** Patients with PD-L1 CPS ≥ 10. **d** Patients with ESCC PD-L1 CPS ≥ 10. Events were defined as patients who died. *CI* confidence interval, *CPS* combined positive score, *ESCC* esophageal squamous cell carcinoma, *HR* hazard ratio, *NE* not evaluable, *PD-L1* programmed death ligand 1

By the time of interim OS analysis, 42 of 74 patients (56.8%) in the pembrolizumab–chemotherapy group and 45 of 67 patients (67.2%) in the placebo–chemotherapy group died; median OS (95% confidence interval [CI]) was 17.6 months (13.9–not evaluable [NE]) and 11.7 months (9.5–19.0), respectively (HR, 0.71; 95% CI 0.47–1.09) (Fig. 1a). The 12-month OS rate was 73.0% for pembrolizumab–chemotherapy and 49.3% for placebo–chemotherapy.

Analysis of OS by subgroup demonstrated prolonged survival with pembrolizumab–chemotherapy compared with placebo–chemotherapy in Japanese patients with ESCC, PD-L1 CPS ≥ 10, and ESCC PD-L1 CPS ≥ 10 (Fig. 1b–d). In the ESCC subgroup, 37 of 67 patients (55.2%) receiving pembrolizumab–chemotherapy and 40 of 59 patients (67.8%) receiving placebo–chemotherapy died (Fig. 1b); median OS (95% CI) was 17.7 months (13.7–NE) and 11.7 months (9.6–18.3), respectively (HR, 0.69; 95% CI 0.44–1.08). The 12-month OS rate was 73.1% for pembrolizumab–chemotherapy and 49.2% for placebo–chemotherapy. In the PD-L1 CPS ≥ 10 subgroup, 29 of 48 patients (60.4%) receiving pembrolizumab–chemotherapy and 28 of 36 patients (77.8%) receiving placebo–chemotherapy died (Fig. 1c); median OS (95% CI) was 16.9 months (13.5–NE) and 11.2 months (7.9–15.4), respectively (HR, 0.58; 95% CI 0.35–0.98). The 12-month OS rate was 72.9% for pembrolizumab–chemotherapy and 41.7% for placebo–chemotherapy. In the ESCC PD-L1 CPS ≥ 10 subgroup, 27 of 44 patients (61.4%) receiving pembrolizumab–chemotherapy and 26 of 32 patients (81.3%) receiving placebo–chemotherapy died (Fig. 1d); median OS (95% CI) was 15.8 months (12.8–NE) and 10.9 months (7.8–14.6), respectively (HR, 0.55; 95% CI 0.32–0.94). The 12-month OS rate was 70.5% for pembrolizumab–chemotherapy and 37.5% for placebo–chemotherapy.

### Progression-free survival

**Fig. 2:**
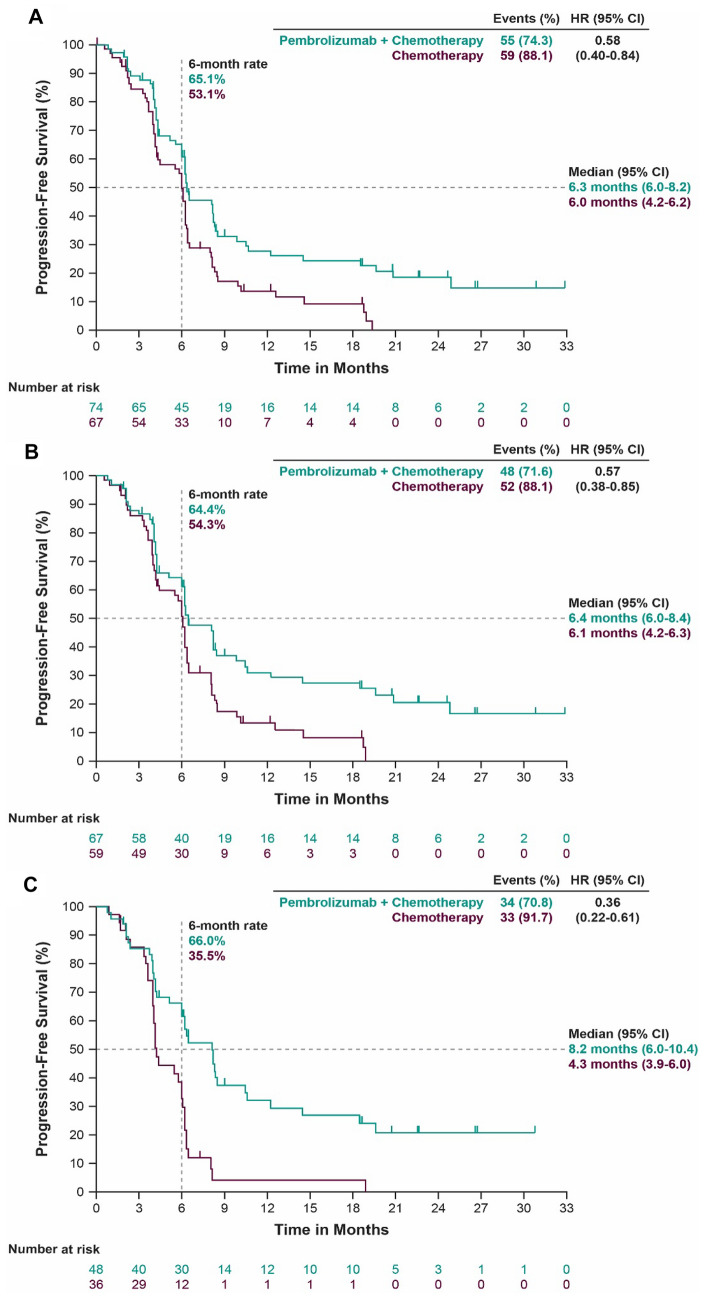
Kaplan–Meier estimates of progression-free survival in the Japanese population by treatment group. **a** All patients. **b** Patients with ESCC. **c** Patients with PD-L1 CPS ≥ 10. Events were defined as patients with progressive disease or patients who died. *CI* confidence interval, *CPS* combined positive score, *ESCC* esophageal squamous cell carcinoma, *HR* hazard ratio, *PD-L1* programmed death ligand 1

By the time of final PFS analysis, 55/74 patients (74.3%) in the pembrolizumab–chemotherapy group and 59/67 patients (88.1%) in the placebo–chemotherapy group died or experienced disease progression (Fig. 2a); median PFS (95% CI) was 6.3 months (6.0–8.2) and 6.0 months (4.2–6.2), respectively (HR, 0.58; 95% CI 0.40–0.84). The 6-month PFS rate was 65.1% for pembrolizumab–chemotherapy and 53.1% for placebo–chemotherapy. Pembrolizumab–chemotherapy was favored for PFS over placebo–chemotherapy in the ESCC and PD-L1 CPS ≥ 10 subgroups (Fig. 2b, c). In the ESCC subgroup, 48 of 67 patients (71.6%) receiving pembrolizumab–chemotherapy and 52 of 59 patients (88.1%) receiving placebo–chemotherapy died or experienced disease progression; median PFS (95% CI) was 6.4 months (6.0–8.4) and 6.1 months (4.2–6.3), respectively (HR, 0.57; 95% CI 0.38–0.85). The 6-month PFS rate was 64.4% for pembrolizumab–chemotherapy and 54.3% for placebo–chemotherapy. In the PD-L1 CPS ≥ 10 subgroup, 34 of 48 patients (70.8%) receiving pembrolizumab–chemotherapy and 33 of 36 (91.7%) receiving placebo–chemotherapy died or experienced disease progression; median PFS (95% CI) was 8.2 months (6.0–10.4) and 4.3 months (3.9–6.0), respectively (HR, 0.36; 95% CI 0.22–0.61). The 6-month PFS rate was 66.0% for pembrolizumab–chemotherapy and 35.5% for placebo–chemotherapy.

### Response

In the Japanese population, 42 of 74 patients (56.8%) in the pembrolizumab–chemotherapy group and 26 of 67 patients (38.8%) in the placebo–chemotherapy group achieved objective response. The median DOR (range) was 8.3 months (1.2 + to 31.0 +) and 6.1 months (3.5–17.4), respectively. In the ESCC subgroup, 38 of 67 patients (56.7%) receiving pembrolizumab–chemotherapy and 24/59 (40.7%) receiving placebo–chemotherapy achieved objective response; median DOR (range) was 10.4 months (1.2 + to 31.0 +) and 6.1 months (3.5–17.0), respectively. In the CPS PD-L1 ≥ 10 subgroup, 29 of 48 patients (60.4%) receiving pembrolizumab–chemotherapy and 11 of 36 patients (30.6%) receiving placebo–chemotherapy achieved objective response; median DOR (range) was 10.4 months (2.3 + to 28.9 +) and 4.4 months (3.5–17.0), respectively. In the ESCC PD-L1 CPS ≥ 10 subgroup, 26 of 44 patients (59.1%) receiving pembrolizumab–chemotherapy and 10 of 32 (31.3%) receiving placebo–chemotherapy achieved objective response; median DOR (range) was 10.5 (2.3 + 28.9 +) and 4.4 months (3.5–17.0), respectively.

### Safety

All patients in the Japanese population experienced ≥ 1 adverse event (AE) (Table 2). Treatment-related AEs (TRAEs) occurred in 73 of 74 patients (98.6%) in the pembrolizumab–chemotherapy group and 66 of 67 patients (98.5%) in the placebo–chemotherapy group (Table 2). Grade 3–5 TRAEs were reported in 55 of 74 patients (74.3%) and 41 of 67 patients (61.2%), respectively. Treatment-related deaths occurred in 2 of 74 patients in the pembrolizumab–chemotherapy group (2.7%; interstitial lung disease and pneumonitis) and 1 of 67 patients in the placebo–chemotherapy group (1.5%; interstitial lung disease). The most common TRAEs in the pembrolizumab–chemotherapy and placebo–chemotherapy groups were decreased appetite (78.4% and 58.2%), nausea (74.3% and 62.7%), and decreased neutrophil count (60.8% and 56.7%) (Table 2). Immune-mediated AEs were reported in 21 of 74 patients (28.4%) in the pembrolizumab–chemotherapy group and 9 of 67 patients (13.4%) in the placebo–chemotherapy group (Table 3). The most common immune-mediated AEs (≥ 5%) in the pembrolizumab–chemotherapy group were hypothyroidism (9.5%), pneumonitis (6.8%), colitis (5.4%), and severe skin reactions (5.4%).

**Table 2:** Adverse events in the Japanese population

Event, *n* (%)	Pembrolizumab + chemotherapy *n* = 74	Placebo + chemotherapy *n* = 67
≥ 1 AE	74 (100)	67 (100)
Grade 3–5	61 (82.4)	49 (73.1)
Led to discontinuation	19 (25.7)	14 (20.9)
Serious	31 (41.9)	32 (47.8)
Serious and led to discontinuation	10 (13.5)	6 (9.0)
Led to death	4 (5.4)	1 (1.5)
≥ 1 treatment-related AE	73 (98.6)	66 (98.5)
Grade 3–5	55 (74.3)	41 (61.2)
Led to discontinuation	15 (20.3)	11 (16.4)
Serious	24 (32.4)	16 (23.9)
Serious and led to discontinuation	7 (9.5)	4 (6.0)
Led to death^a^	2 (2.7)	1 (1.5)
Treatment-related AEs occurring in ≥ 20% of patients in either group		
Decreased appetite	58 (78.4)	39 (58.2)
Nausea	55 (74.3)	42 (62.7)
Decreased neutrophil count	45 (60.8)	38 (56.7)
Stomatitis	42 (56.8)	35 (52.2)
Decreased white blood cell	35 (47.3)	22 (32.8)
Anemia	28 (37.8)	29 (43.3)
Fatigue	26 (35.1)	10 (14.9)
Malaise	26 (35.1)	23 (34.3)
Constipation	23 (31.1)	19 (28.4)
Hiccups	23 (31.1)	15 (22.4)
Increased blood creatine	20 (27.0)	22 (32.8)
Diarrhea	20 (27.0)	18 (26.9)
Alopecia	18 (24.3)	13 (19.4)
Decreased platelet count	17 (23.0)	14 (20.9)
Dysgeusia	16 (21.6)	13 (19.4)
Peripheral sensory neuropathy	15 (20.3)	14 (20.9)
Hyponatremia	10 (13.5)	16 (23.9)

*AE* adverse event

^a^Two patients in the pembrolizumab–chemotherapy group died of treatment-related interstitial lung disease and pneumonitis and 1 patient in the placebo-chemotherapy group died of interstitial lung disease

**Table 3:** Immune-mediated adverse events and infusion reactions in the Japanese population

Event, *n* (%)	Pembrolizumab + chemotherapy *n* = 74	Placebo + chemotherapy *n* = 67
Hypothyroidism	7 (9.5)	5 (7.5)
Pneumonitis	5 (6.8)	1 (1.5)
Colitis	4 (5.4)	1 (1.5)
Severe skin reactions	4 (5.4)	0
Hyperthyroidism	3 (4.1)	1 (1.5)
Hypophysitis	2 (2.7)	0
Infusion reactions	2 (2.7)	1 (1.5)
Adrenal insufficiency	1 (1.4)	2 (3.0)
Hepatitis	1 (1.4)	0
Type 1 diabetes mellitus	1 (1.4)	0
Nephritis	0	1 (1.5)

## Discussion

Poor prognosis and limited treatment options highlight the unmet need in Japanese patients with advanced EC. In this subgroup analysis of Japanese patients enrolled in KEYNOTE-590, first-line pembrolizumab–chemotherapy prolonged OS and PFS and improved response rates over placebo–chemotherapy. The safety profile was comparable between treatment groups, and no new safety signals were detected. These findings in Japanese patients are consistent with the data reported in the total population of KEYNOTE-590 [9].

Use of a two-drug cytotoxic chemotherapy regimen for first-line treatment in Japanese patients with advanced EC has shown response rates of 20–60% [3]. However, median survival times remain low, as described in a retrospective analysis of cisplatin plus 5-FU treatment in which the median OS was 10.4 months [10]. Three-drug chemotherapy regimens have demonstrated improved response rates compared with two-drug regimens; however, they are associated with higher toxicities, and the impact on survival is unknown [3, 11, 12]. Targeted therapy with trastuzumab has improved clinical outcomes in HER2-positive gastric or gastroesophageal cancer when added to first-line chemotherapy and is included in National Comprehensive Cancer Network and Pan-Asian European Society for Medical Oncology guidelines [11, 13, 14]. Other targeted therapies have not been successful in improving clinical outcomes as first-line treatment when combined with chemotherapy for advanced EC [15, 16].

Data with first-line anti–PD-1/PD-L1 treatment in patients with EC are limited but promising. Pembrolizumab and nivolumab have demonstrated significant improvements in survival with second-line treatment in advanced EC [6, 17]. In KEYNOTE-181, pembrolizumab prolonged OS compared with chemotherapy in patients with ESCC PD-L1 CPS ≥ 10 (HR, 0.64; 95% CI 0.46–0.90) [6, 18]. Pembrolizumab is approved in the United States and Japan for second-line treatment of patients with ESCC PD-L1 CPS ≥ 10 [5, 18]. In ATTRACTION-3, nivolumab significantly improved OS compared with chemotherapy in patients with ESCC (HR, 0.77; 95% CI 0.62–0.96; *P* = 0.019) [17]. Based on these data, nivolumab is approved in the United States and Japan for second-line treatment of patients with ESCC [19]. Recent results from the KEYNOTE-590 primary analysis (ESCC and adenocarcinoma) and CheckMate-649 (gastric cancer and esophageal adenocarcinoma) studies have demonstrated that first-line treatment with immune checkpoint inhibitors has the potential to be standard of care for patients with EC. In KEYNOTE-590, pembrolizumab–chemotherapy was superior to placebo–chemotherapy for OS (HR, 0.73; *P* < 0.0001) and PFS (HR, 0.65; *P* < 0.0001) in patients with unresectable locally advanced metastatic esophageal adenocarcinoma or ESCC or Siewert type I EGJ adenocarcinoma [9]. In CheckMate-649, nivolumab plus chemotherapy was compared with chemotherapy as first-line treatment of patients with unresectable or metastatic gastric cancer, gastroesophageal junction cancer, or esophageal adenocarcinoma; patients with ESCC were not included in this study [20]. In data presented thus far, the benefit of nivolumab plus chemotherapy for patients with esophageal cancer, regardless of histology, is unclear.

In this analysis from KEYNOTE-590, pembrolizumab–chemotherapy improved clinical outcomes compared with placebo–chemotherapy in the Japanese population. Although subpopulation sample sizes were small in the Japanese population, clinical outcomes were further improved in patients with ESCC, PD-L1 CPS ≥ 10, and ESCC ﻿PD-L1 CPS ≥ 10 compared with the overall Japanese population. Median OS in the pembrolizumab–chemotherapy group was longer in the Japanese population than in the total population [9]. Median OS and PFS were 17.6 and 6.3 months in the Japanese population and 12.4 and 6.3 months in the total population; the higher percentage of patients with ESCC and ﻿PD-L1 CPS ≥ 10 receiving subsequent systemic therapy in the Japanese population may explain this result. Most patients in the pembrolizumab–chemotherapy (68.8%) and the placebo–chemotherapy (75.4%) groups received subsequent systemic therapy; nonetheless, pembrolizumab–chemotherapy-treated patients experienced clinically meaningful improvement in OS compared with placebo–chemotherapy-treated patients. A higher percentage of patients in the Japanese population than in the total population received subsequent therapy (pembrolizumab–chemotherapy, 68.8% vs. 43.5%; placebo–chemotherapy, 75.4% vs. 47.8%), potentially contributing to improved outcomes in the pembrolizumab group in the Japanese population. Although the study was double-blind, patients received immune checkpoint inhibitor therapy at a lower rate in the pembrolizumab–chemotherapy group (10.9%) than in the placebo–chemotherapy group (16.9%), but this did not appear to have an impact on clinical outcomes. Notably, a higher proportion of Japanese patients had better ECOG performance status at baseline in both the pembrolizumab–chemotherapy group and the placebo–chemotherapy group (ECOG performance status  0, 64.9% and 79.1%, respectively) compared to the same groups in the total population (ECOG performance status  0, 40% and 40%, respectively) [7], which could have contributed to the higher proportion of Japanese patients who received subsequent therapy. Responses to pembrolizumab–chemotherapy were durable in Japanese patients and in line with the total population (median DOR, 8.3 months).

The safety profile of pembrolizumab–chemotherapy was consistent between Japanese patients and the total population [9]. Treatment-related AEs were reported in 98.6% patients in the Japanese population versus 98.4% in the total population; grade 3–5 treatment-related AEs were reported in 74.3% versus 71.9% patients, respectively.

A limitation of this study is the evaluation of a subgroup of patients from a larger clinical trial in which Japanese patients represented approximately 20% of the total population. Given that 90% of the Japanese population had ESCC, evaluation of clinical outcomes in the overall Japanese population may not be directly comparable to those in the total population (73% ESCC). Small sample sizes in the ESCC, PD-L1 CPS ≥ 10, and ESCC PD-L1 CPS ≥ 10 Japanese subgroups limit conclusions about the improved outcomes with pembrolizumab–chemotherapy versus placebo–chemotherapy.

Pembrolizumab–chemotherapy improved clinical outcomes, including OS, PFS, and ORR, compared with placebo–chemotherapy in Japanese patients with treatment-naive advanced EC. The safety profile was comparable between the two treatment groups, and there were no new safety signals for pembrolizumab in the Japanese population. These data suggest that pembrolizumab–chemotherapy should be considered a new first-line treatment option for all Japanese patients with unresectable recurrent or advanced EC.

## Supplementary Information

Below is the link to the electronic supplementary material.

Supplementary file1 (PDF 312 KB)

## Data Availability

Data collection was provided by each clinical trial site and data were analyzed by the authors. All authors had full access to the data and take responsibility for the integrity of the data and the accuracy of the data analysis. Medical writing and/or editorial assistance was funded by Merck Sharp & Dohme LLC, a subsidiary of Merck & Co., Inc., Rahway, NJ, USA.
