# Supplementary material for: First-line pembrolizumab + chemotherapy in Japanese patients with advanced/metastatic esophageal cancer from KEYNOTE-590
Source: Esophagus. 2022 Jun 7;19(4):683–92. doi: 10.1007/s10388-022-00920-x (PMC9436840; doi:10.1007/s10388-022-00920-x)
Supplement: Supplementary file 1 — Supplementary file1 (PDF 312 KB) [file 10388_2022_920_MOESM1_ESM.pdf]

Online supplemental material

First-line pembrolizumab + chemotherapy in Japanese patients with advanced/metastatic esophageal cancer from KEYNOTE-590

Takashi Kojima[a], Hiroki Hara[b], Akihito Tsuji[c], Hisateru Yasui[d], Kei Muro[e], Taroh Satoh[f], Takashi Ogata[g], Ryu Ishihara[h], Masahiro Goto[i], Hideo Baba[j], Tomohiro Nishina[k], Shirong Han[l], Tomoko Sakata[l], Naoyoshi Yatsuzuka[l], Toshihiko Doi[m], Ken Kato[n]

[a]Department of Gastroenterology and Gastrointestinal Oncology, National Cancer Center Hospital East, Chiba, Japan; [b]Department of Gastroenterology, Saitama Cancer Center, Saitama, Japan; [c]Department of Medical Oncology, Kagawa University Hospital, Kagawa, Japan; [d]Department of Medical Oncology, Kobe City Medical Center General Hospital, Hyogo, Japan; [e]Department of Clinical Oncology, Aichi Cancer Center Hospital, Aichi, Japan; [f]Department of Frontier Science for Cancer and Chemotherapy, Osaka University Hospital, Osaka, Japan; [g]Department of Gastrointestinal Surgery, Kanagawa Cancer Center, Kanagawa, Japan; [h]Department of Gastrointestinal Oncology, Osaka International Cancer Institute, Osaka, Japan; [i]Division of Medicine, Osaka Medical College Hospital, Osaka, Japan; [j]Department of Gastroenterological Surgery, Kumamoto University Hospital, Kumamoto, Japan; [k]Department of Gastrointestinal Medical Oncology, National Hospital Organization Shikoku Cancer Center, Ehime, Japan; [l]Department of Medical Oncology, MSD K.K., Tokyo, Japan; [m]Department of Gastrointestinal Oncology, National Cancer Center Hospital East, Chiba, Japan; [n]Department of Gastrointestinal Medical Oncology, National Cancer Center Hospital, Tokyo, Japan

Esophagus

Corresponding Author
Ken Kato, MD, PhD
National Cancer Center Hospital
5-1-1, Tsukiji, Chuo-ku
Tokyo, 104-0045, Japan
Phone: 03-3542-2511 (7902)
Fax: 03-3452-3815
Email: kenkato@ncc.go.jp

Online Resource 1. CONSORT diagram.

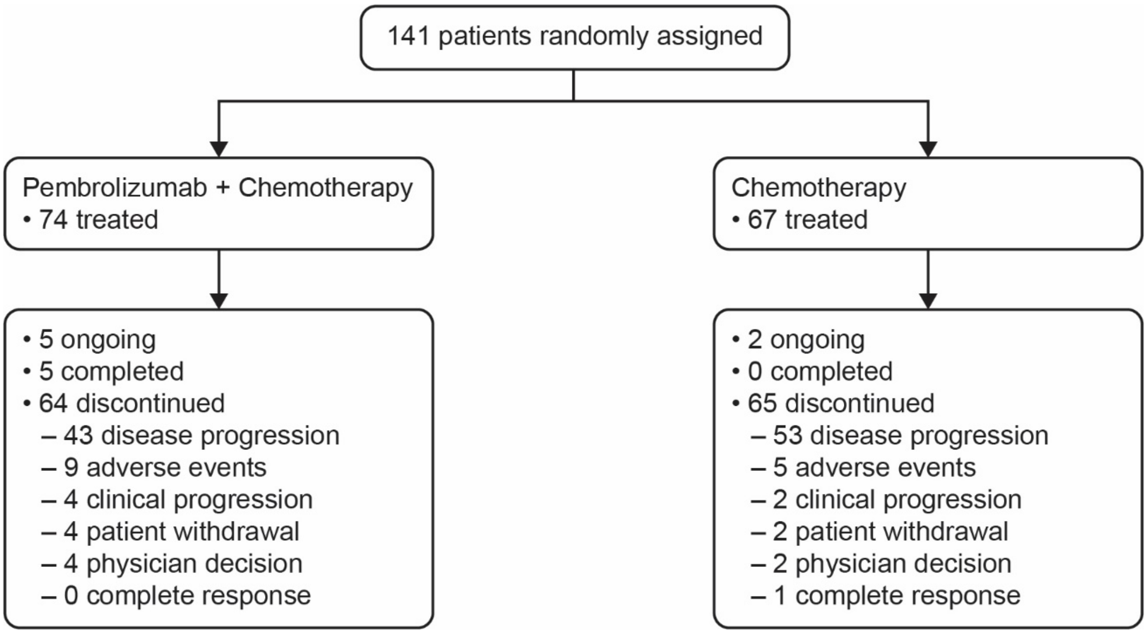

Online Resource 2. Subsequent therapy in the Japanese population

**Therapy, n (%)**	**Pembrolizumab + chemotherapy** ***n* = 64[a]**	**Chemotherapy** ***n* = 65[a]**
**All patients**	44 (68.8)	49 (75.4)
**Incidence ≥5% in any treatment group**		
**Cisplatin**	11 (17.2)	12 (18.4)
**Docetaxel**	5 (7.8)	8 (12.3)
**Fluorouracil**	20 (31.3)	14 (21.5)
**Oxaliplatin**	10 (15.6)	2 (3.1)
**Paclitaxel**	31 (48.4)	36 (55.4)
**Ramucirumab**	5 (7.8)	4 (6.2)
**Immunotherapy incidence ≥0%**		
**Anti–LAG-3 mAb**	0 (0)	1 (1.5)
**Anti–PD-1 mAb**	0 (0)	1 (1.5)
**Nivolumab**	5 (7.8)	11 (16.9)
**Pembrolizumab**	2 (3.1)	0 (0)

[a]Number of patients who discontinued study medication.
